# Core encoding sequences of Hepatitis C virus in Ghanaian blood donors are predominantly mosaics of different genotype 2 strains and cannot distinguish subtypes

**DOI:** 10.1186/s12879-019-4155-4

**Published:** 2019-06-17

**Authors:** Nicholas Israel Nii-Trebi, Charles Addoquaye Brown, Yaa Difie Osei, William Kwabena Ampofo, Alexander Kwadwo Nyarko

**Affiliations:** 1 Department of Medical Laboratory Sciences, School of Biomedical and Allied Health Sciences, College of Health Sciences, University of Ghana, Accra, Ghana; 2 Department of Biochemistry, Cell and Molecular Biology, School of Biological Sciences, College of Basic and Applied Sciences, University of Ghana, Accra, Ghana; 3 Department of Virology, Noguchi Memorial Institute for Medical Research, College of Health Sciences, University of Ghana, Accra, Ghana; 4 Department of Clinical Pathology, Noguchi Memorial Institute for Medical Research, College of Health Sciences, University of Ghana, Accra, Ghana

**Keywords:** HCV, Seroprevalence, Genotype, Core gene, Molecular epidemiology, Blood donors, Ghana

## Abstract

**Background:**

Distribution of Hepatitis C virus (HCV) genotypes varies significantly worldwide. Genomic diversity between genotypes has implications for treatment, vaccine development and optimal design of HCV diagnostic assays. Molecular characterization of HCV in different geographical areas is therefore very essential for management and public health control of HCV infection. This study investigated the molecular epidemiology and characteristics of HCV genotypes in healthy individuals in Accra, Ghana.

**Methods:**

An experimental study was carried out on blood samples obtained from voluntary blood donors. Two hundred samples were initially screened for HCV antibodies and infection was confirmed by RNA detection through RT-PCR of the 5′-untranslated region (5’UTR). The core gene sequences were analysed for HCV genotype determination by genotype-specific PCR; and then by cloning and direct sequencing followed by phylogenetic analysis. The sequences were further analysed in detail by similarity plotting.

**Results:**

Molecular diagnosis confirmed the presence of HCV RNA in 2 out of 200 (1%) blood donors. Initial genotyping by genotype-specific PCR identified all two infections as subtypes 2a and 2b of genotype 2. Extensive evolutionary and genetic analyses indicated two epidemiological profiles. First, phylogenetic tree topologies clearly showed that, collectively, the core sequences of the Ghanaian HCV isolates belong to a single, distinct genetic group within HCV genotype 2 cluster, with high genetic similarity and rapid sequence variation in a single individual. Second, the sequences are mosaics comprising 2e and other genotype 2 subtype fragments. The analyses underscore a unique and complex HCV genotype 2 core sequence profile of the Ghanaian isolates.

**Conclusions:**

Analysis of HCV core encoding sequences from Ghanaian blood donors in Accra confirmed predominance of genotype 2 HCV among healthy individuals. However, the isolates could not be classified into subtypes, possibly due to their complex sequence pattern that might suggest high mutability of the prevailing genotype. The core region of Ghanaian HCV therefore may not be suitable for distinguishing subtypes. These findings extend those from previous studies and thus underscore the need to search for subtype-informative region of Ghanaian HCV to elucidate the genetic diversity and factors determining outcome of HCV infections in Ghana.

## Background

Viral hepatitis presents a major health problem throughout the world [1]. Hepatitis C virus (HCV) is the most important agent of chronic or acute parenterally transmitted viral hepatitis [2]. The virus is able to establish persistent infections in more than 90% of infected people and cause quiescent, progressive and asymptomatic infections for several years [3, 4]. Its replication is characterized by high rate of genetic mutation, as a result of lack of fidelity of the RNA-dependent RNA polymerase encoded by the non-structural 5B (NS5B) gene [5–7]. High genetic mutation of the HCV genome results in rapid generation of viral variants. Even within an infected individual HCV variants emerge having genomes that are either very closely related, referred to as quasispecies, or genetically distinct, referred to as genotypes or subtypes [8–10].

Previously, HCV was classified into six phylogenetically distinct genotypes numbered 1–6 [11, 12]. Subsequently, comparison of sequences derived from various geographical locations have shown that a large number of HCV variants exist [13, 14]. Recently, analysis of over 1300 (near-)complete HCV genome sequences available on public databases using consensus criteria classified HCV into seven confirmed genotypes (numbered 1 to 7) and 67 subtypes; and identified 21 additional complete coding region sequences of unassigned subtype [15]. Molecular epidemiology studies have shown that these HCV genotypes and subtypes are differently distributed in different regions of the world, and certain genotypes predominate in certain regions [16, 17].

Africa is reported as the World Health Organization (WHO) region with the highest HCV prevalence. The average prevalence estimated for sub-Saharan Africa is approximately 3.0% [18–20]; and there exist extensive diversity within genotypes in Central and West African countries [21–24]. Importantly, genotypes 1 and 2 are suggested to be endemic in West Africa [25, 26] where the present study country, Ghana, is located. A recent comprehensive, systematic review by Akosua Agyeman and colleagues [27] estimated the prevalence of chronic HCV infection among Ghanaian blood donors as 2.6%. Other studies also confirmed the predominance of genotype 2 HCV [22, 28]. However, the prevailing HCV subtypes in the country are yet to be completely determined. Recently, a Bayesian phylogeographic analysis using discrete trait model showed Ghana as the most likely geographic region for the origin of HCV genotype 2 [29]. Candotti and colleagues previously analysed the E1/E2 and NS5 regions; and, noting the challenge in distinguishing genotype 2 subtypes, have described the Ghanaian HCV sequences as diverse. Notwithstanding, other studies have found the core encoding region suitable and ideal for HCV classification at the subtype level [30, 31]. This study therefore aimed to describe the genetic diversity of prevailing HCV genotypes in healthy individuals in Accra, Ghana, based on analysis of the core encoding region. Analysis showed that the core sequences of the HCV isolates detected were complex and phylogenetically distinct from other HCV 2 subtypes. This study thus reports that the core gene of Ghanaian HCV isolates may not be suitable for distinguishing HCV genotype 2 subtypes.

## Methods

### Samples

This study was cross-sectional and experimental. It was carried out on 200 consenting voluntary blood donors sampled through a national blood donation exercise in Accra. Random samples were collected in four millilitres EDTA tubes (Vacutainer Systems, Frankh Lakes, N. J., USA). Within 2 h of drawing blood, plasma was separated after centrifugation and immediately frozen at –70 °C until used for serological testing and virus RNA extraction.

### HCV antibodies testing and confirmation

Plasma from donor specimens were serologically screened and confirmed for the presence of anti-HCV antibodies*.* Initial *s*creening was done with SERODIA-HCV particle agglutination (PA) assay (FUJIREBIO INC., Tokyo, Japan). All sera, both PA reactive and nonreactive were retested with Murex anti-HCV version 4.0 Enzyme-linked Immunosorbent Assay (ELISA) (Murex Biotech SA Ltd., Kyalami, South Africa). All assays were performed strictly according to the manufacturers’ instructions. HCV RNA extraction and amplification were performed on sera that were reactive by either PA or anti-HCV EIA; and PCR-confirmed specimens were genotyped.

### RNA preparation and PCR amplification

Total RNA was extracted from 100 μl of plasma by SepaGene RNA extraction kit (Sanko Junyaku Co. Ltd., Tokyo, Japan) according to the manufacturer’s instructions. Extracted RNA were vacuum-dried and resuspended in 40 μl of diethyl pyrocarbonate (DEPC)-treated water (DEPC was by SIGMA Chemical Co., Steinheim, Germany); and stored at –70 °C until use. Extracted RNA was reverse-transcribed with Moloney murine leukemia virus (M-MuLV) reverse transcriptase and cDNA amplification was by AmpliT*aq* Gold DNA polymerase with primers located in the highly conserved 5′-untranslated region (5’UTR) as described below.

### HCV RNA detection

RT-PCR was performed by a one-step method using Ready-To-Go RT-PCR bead system (Amersham Pharmacia Biotech Inc., N. J, USA) optimized for first strand cDNA synthesis and PCR. The RT-PCR reaction was carried out in a final volume of 50 μl containing 10 units of RNAguard, 100 units of M-MuLV reverse transcriptase, 200 μM of each deoxynucleotide triphosphate (dNTP), 2 units of AmpliT*aq* Gold DNA polymerase and 1.5 mM MgCl_2_ in RNase-free water, to which 0.65 μl of 10 μM each HCV-specific outer primer and 5 μl of RNA template were added. Globin mRNA was used as HCV positive control. Previously described primers, HCV 19 (GCGACACTCCACCATAGAT) and HCV 20 (GCTCATGGTGCACGGTCTA [32] were used for the RT-PCR. These yielded a fragment of 329 bp. Reverse transcription was achieved by incubating the mixture at 42 °C for 30 min, followed by AmpliT*aq* Gold DNA polymerase activation step at 95 °C for 5 min. First round PCR comprised 40 cycles with the following parameters: denaturation at 94 °C for 30 s, annealing at 50 °C for 45 s, and extension at 72 °C for 1 min. The reverse transcription and amplification reactions were done using Perkin-Elmer GeneAmp PCR System 2400 (Norwalk CT, USA).

Second round PCR was carried out using Ready-To-Go PCR reaction system. Bead in the reaction tube was dissolved in 22.3 μl RNase-free water; 0.35 μl each of the pair of inner primers HCV 21/HCV22 [32] and 2 μl of first round PCR product were added. When brought to a final volume of 25 μl, the reaction mixture contained 1.5 units of T*aq* DNA Polymerase, 10 mM Tris-HCl (pH 9.0 at room temperature), 50 mM KCl, 1.5 mM MgCl_2_ and 200 μM of each dNTP. Nested PCR conditions comprised preheating at 94 °C for 2 mins, followed by two 20-cycle rounds of PCR. Conditions for the first 20 cycles of amplification comprised denaturation at 94 °C for 30 s, annealing at 53 °C for 45 s and extension at 72 °C for 1 min. Conditions for the second 20 cycles round of amplification comprised 94 °C for 30 s, 55 °C for 45 s and 72 °C for 1 min. The expected product size was 268 bp. PCR products were evaluated by electrophoresis in 2% agarose gel in 1X Tris-Acetic Ethylenediaminetetraacetic acid (1X TAE). Tris base (Tris[hydroxymethyl]aminomethane), EDTA and ethidium bromide were obtained from SIGMA Chemical Co., Steinheim, Germany; agarose was from Invitrogen, Life Technologies, Paisley, Scotland.

### HCV genotyping

HCV PCR positive samples were genotyped by type-specific PCR using primers that selectively amplify different genotypes as described by Ohno et al. [33]. Briefly, the core region of the HCV genome was reverse-transcribed, and two rounds of PCR were performed. PCR amplification was done using Ready-To-Go RT-PCR. RT-PCR reaction bead containing M-MuLV reverse transcriptase. The bead was dissolved in 40.5 μl of RNase-free water; 2.5 μl of 1 μg/ul random hexamer pd.(N)_6_ (GIBCO BRL, Gaithersburg, Md.) and 5 μl RNA template was added. RNA reverse-transcription was achieved by incubating the reaction mixture at 42 °C for 25 mins and heating at 95 °C for 5 mins. First round PCR utilized primers Sc2 and Ac2 [33], and T*aq* DNA Polymerase. One microliter of each 2.5 μM primer was added to the RT reaction tube. Amplification conditions included pre-incubation at 94 °C for 1 min, followed by 40 cycles with the following parameters: a preliminary 20 cycles amplification through denaturation at 94 °C for 1 min, annealing at 45 °C for 1 min and extension at 72 °C for 1 min; followed by additional 20 cycles of 94 °C for 1 min, 60 °C for 1 min and 72 °C for 1 min.

Two different second-round PCRs were performed for each sample by the method of Ohno et al. [33] modified for use in a Ready-To-Go PCR bead system. Briefly, two different primer mixtures were prepared per sample - one containing S7, S2a, G1b, G2a, G2b and G3b primers (Mix 1) and another containing S7, G1a, G3a, G4, G5a and G6a primers (Mix 2). The total reaction volume was 25 μl and made up of 18.5 μl RNase-free water, 6 μl of 2.5 μM PCR primer Mix 1 or Mix 2 and 0.5 ul of first-round PCR amplicon as template. Amplification was performed by preheating at 94 °C for 1 min, followed by 30 cycles of reaction. Each cycle comprised denaturation at 94 °C for 1 min, annealing at 62 °C for 45 s and extension at 72 °C for 1 min. Ten microliters of the second-round PCR product was evaluated in 2% agarose gel electrophoresis. HCV genotype was determined by identifying genotype-specific DNA bands in gel by their expected sizes based on primers used [33].

### Cloning and sequencing

To verify HCV genotype and determine possible inter- and intra-patient subtype differences, HCV core amplicons (approximately 429 bp) were further analysed. A semi-nested PCR was performed with Sc2 and Ac2 as first-round primers and S7 and Ac2 as second-round primers [33] . The PCR conditions were as described for genotyping above. PCR products were purified from 2% agarose gel using QIAquick gel purification protocol (Qiagen Ltd., Germany) according to the manufacturer’s instructions. Purified amplicons were cloned directly into pCR 2.1-TOPO plasmid vector (~ 3.9 kb) and used to transform chemically competent *Escherichia coli*. Positive clones were detected through purification by Miniprep protocol (Qiagen Ltd.) and digestion with *Eco RI*. LB Agar, LB Broth Base, pCR 2.1 TOPO vector and *Escherichia coli* were obtained from Invitrogen, Life Technologies, Paisley, Scotland; and *Eco RI* was from Roche Diagnostics GmbH., Mannheim, Germany.

For each isolate, at least two clones were sequenced on both strands using BigDye Terminator Cycle Sequencing Ready Reaction kit (Applied Biosystems). Sequencing products were purified by ethanol precipitation protocol. Electrophoresis and data acquisition were done on an automated ABI PRISM 310 genetic analyser (Applied Biosystems). Consensus nucleotide sequences obtained from the isolates were used in phylogenetic analysis.

### Phylogenetic relationships and evolutiona**r**y analyses

Nearly complete core coding sequence (420 nt) corresponding to positions 342–761 of H77 reference sequence [accession number AF009606] was analysed. Representative sequences from the 7 different HCV clades were selected from the 2015 updated alignment of HCV genotypes and subtypes provided by the International Committee on Taxonomy of Viruses (ICTV), available at https://hcv.lanl.gov/content/sequence/NEWALIGN/align.html/ (the ICTV website). More genotype 2 sequences from Ghana were included for analysis to clarify phylogenetic relationships. Reference and test viral core sequences were first aligned using CLUSTAL W program and edited using BioEdit version 7.2.5 [34]. Subsequently, multiple sequence alignment was performed by using MUSCLE program implemented in Molecular Evolutionary Genetics Analysis (MEGA) software [35] (megasoftware.net/index.html); and evolutionary distances were determined based on the maximum likelihood model. Phylogenetic trees with all branch lengths drawn to scale were constructed by neighbour-joining algorithm modelled with Kimura-2 parameter method set for 1000 bootstrap replicates; and values above 700 (70%) were considered to support branching clusters. All phylogenetic relationships and molecular evolutionary analyses were conducted using MEGA version 7.

To clarify sequence relationships, similarity plotting and bootscanning were performed using SimPlot software version 3.5.1 [36] with window and step sizes of 300 and 20 nucleotides respectively. Representative sequences of all genotype 2 subtypes so far described, and other genotype 2 sequence from Ghana, were used as references in the SimPlot analyses.

### Sequence repository

All sequences have been deposited with DNA Databank of Japan (DDBJ) under accession numbers LC271214 to LC271217).

### List of reference sequences

Reference sequences retrieved and used for comparison with those obtained in this study are listed by genotype as follows: genotype 1: GenBank nos. HQ537007, AF009606, D90208, D14853, KJ439768; genotype 2: JF735116, D00944, AB047639, HQ639944, AB031663, AB030907, AB661382, D50409, D10988, JF735114, JF735120, KC844042, DQ155561, HM777359, AB031663, JF735111, FN666429, JF735115, KC197238 and JF735112, KJ642629 (GH02), KJ642628 (GH03), KJ642625 (GH06), KJ642623 (GH08); genotype 3: JF735124, D17763 and KJ470619; genotype 4: FJ025854, Y11604, FJ462435, FJ462436 and DQ418786; genotype 5: AF064490 and Y13184; genotype 6: DQ278893, D84262 and Y12083; and genotype 7: EF108306

## Results

### Serological testing, confirmation and genotyping of HCV

**Fig. 1:**
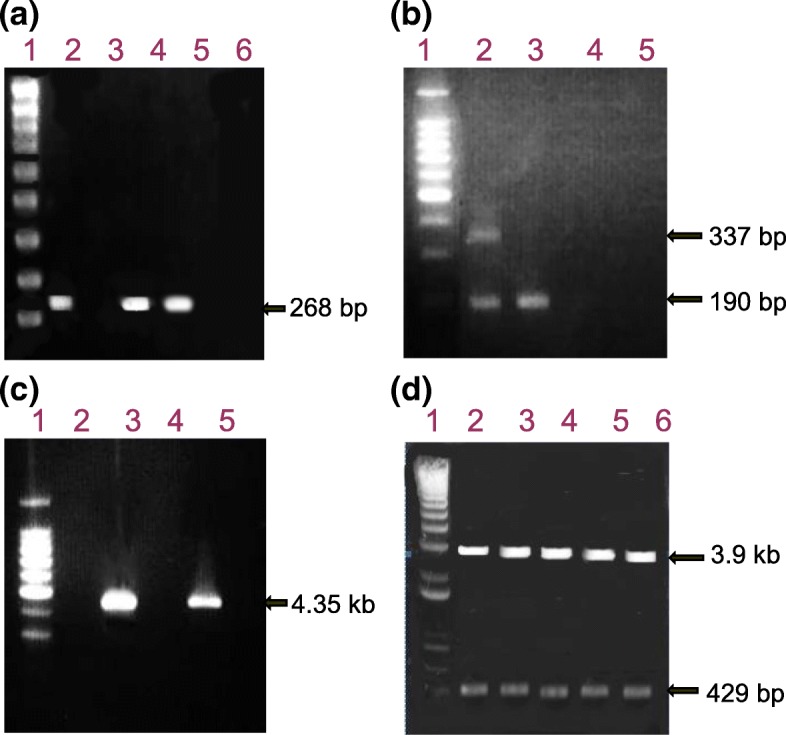
Electrophoretic mobility patterns of PCR products from HCV detection, genotyping and (pre)cloning reactions In all gels first lane displays the molecular weight marker - 100 bp in ‘A’ and ‘B’ and 1 kb in ‘C’ and ‘D’. (**a**) HCV RNA detection in plasma samples. Lane 2 shows cDNA band (268 bp) of globin mRNA used as HCV positive control. Lanes 4 and 5 show 268 bp product indicating presence of HCV RNA in corresponding samples, HCV 152 and HCV 173. (**b**) Primer-specific genotyping of HCV 152 (lane 2) and HCV 173 (lane 3) showing migration positions of HCV genotypes 2a (190 bp) and 2b (337 bp). Two DNA bands with sizes corresponding to genotypes 2a and 2b observed in sample from donor labelled HCV 152 indicated this donor had dual genotype infection. (**c**) Semi-nested PCR amplification products (429 bp) from positive samples (HCV 152 and 173) purified for cloning and sequencing. (**d**) Five products [(HCV 173–1, 173–2, HCV 152–4, 152–5 and 152–8 of Eco RI digested rDNA (4.35 kb) clones purified from *E. coli* cultures. Positive clones showed two bands of sizes 3.9 kb and 429 bp corresponding to the vector and insert cDNA respectively

Two hundred blood samples from voluntary blood donors in Accra were obtained for the study. Initial screening by particle agglutination assay at high plasma dilution (1 in 512) detected 12 (6%) reactive cases. Further testing on ELISA found only 1 (named HCV 152) of the 12 samples as positive; and in all, PCR detected HCV RNA in 2 cases (HCV 152 and HCV 173) out of those pretested by particle agglutination and ELISA. The electrophoretic patterns of PCR products from different reactions are shown in Fig. 1.

PCR-confirmed samples (Fig. 1a) were each analysed twice for genotyping by primer-specific PCR using two primer sets named ‘Mix 1’ and ‘Mix 2’ as described in the methods. Primer ‘Mix 1’ amplification allows for the detection of HCV genotypes 1b, 2a, 2b and 3b; while primer ‘Mix 2’ was for the detection of HCV genotypes 1a, 3a, 4, 5a and 6a. Electrophoresis of the primer-specific genotyping amplification products (Fig. 1b) showed two specific bands of sizes 190 and 337 corresponding to genotypes 2a and 2b respectively in sample HCV 152 (lane 2), whereas one 190 bp band, corresponding to genotype 2a, was observed in HCV 173 (lane 3), all in primer ‘mix 1’ reaction. No bands were seen in the primer ‘Mix 2’ reaction. For cloning and transformation, semi-nested PCR amplification of the HCV core region yielded an expected 429 bp fragment (Fig. 1c). Figure 1d shows digested products of the cloned DNA which were then sequenced for further characterization.

### Phylogenetic characterization of Ghanaian HCV strains

Further molecular characterization of HCV was performed through cloning of confirmed seroreactive HCV isolates. To determine intra- and inter-patient sequence variability, two clones from each sample were selected and sequenced. The clones were named HCV 173_1, 173_2, HCV 152_4 and HCV 152_8 (Fig. 1c). Consensus sequences were obtained and aligned, together with 39 reference sequences representing all available HCV clades (HCV genotypes 1 to 7), and four previously reported Ghanaian HCV isolates.

**Fig. 2:**
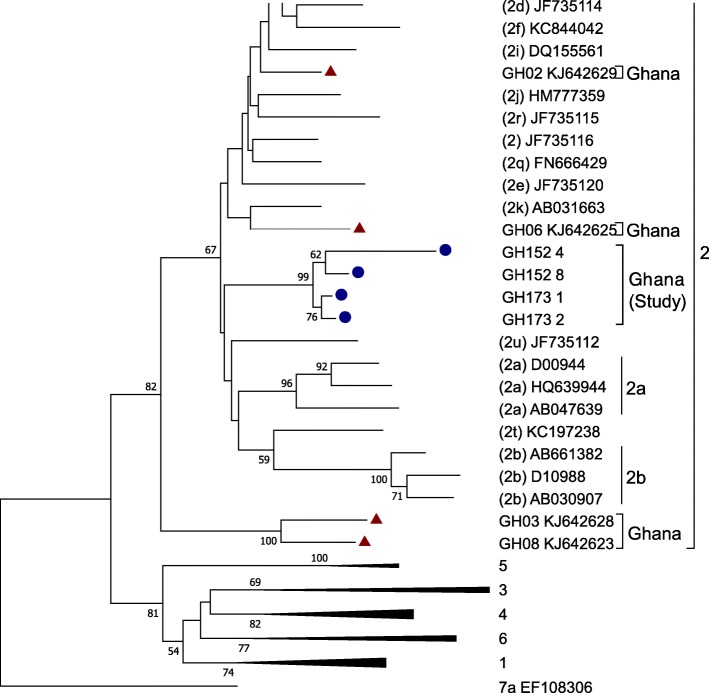
Evolutionary relationship analyses of Ghanaian HCV isolates Phylogenetic analyses of HCV core-encoding region, performed by comparison of Ghanaian isolates with representative nucleotide sequences from all seven clades. The evolutionary history was inferred using neighbour-joining method; and the evolutionary distances were computed using Kimura 2-parameter method. Bootstrap scores were calculated from 1000 replicate analyses and values exceeding 700 (70%) are shown at tree nodes. The tree displays Ghanaian HCV isolates clustered under genotype 2, which are represented by coloured circles (blue); and subtype reference isolates, represented by the subtype (summarized to display only genotype, in the case of 1, 3, 4, 5 and 6) and isolate name. The scale represents number of nucleotide substitutions per site. HCV genotype 7a isolate EF108306 was used as outgroup. Evolutionary analyses were conducted in MEGA7. The branch leading to the group of Ghanaian isolates is 100% supported. The analyses show that the Ghanaian HCV isolates belong to single, unique phylogenetic group within HCV genotype 2 cluster. GH - Ghana

Sequence alignment and phylogenetic analysis showed poor (< 70%) intra-patient sequence homology in one case, HCV 152, which was found to have dual genotype of 2a and 2b by primer-specific genotyping. Three of the isolates - HCV 173_1, 173_2 and HCV 152_8, displayed remarkably high sequence homology, with bootstrap scores ranging from 90 to 100%. HCV 152_4 however exhibited very rapid sequence variation, as compared with the other Ghanaian isolates, having less than 70% sequence homology with the other Ghanaian strains. All the Ghanaian isolates clustered with genotype 2 reference sequences, with bootstrap values of 100% over 1000 replicates (Fig. 2). However, these strains did not clearly cluster with any of the genotype 2 subtypes such as 2a and 2b as was found by primer-specific genotyping, but rather formed a distinct cluster under genotype 2.

To verify whether the sequences from Ghana belong or not to a single genetic group distinct from other African isolates, attempt was made to include more genotype 2 sequences from Ghana and neighbouring countries. Detailed search showed that most sequences described for genotype 2 were either based on NS5B and E1/E2 genes or are too short for meaningful genetic comparison. Four HCV core sequences from Ghana, each of about 348 bp (365–712 of H77 reference sequence numbering), were obtained and included for genetic comparison. At least two of these – GH03 and GH08 showed similar unique phylogenetic clustering. These are identified by GH numbers against their accession numbers (in the list of reference sequences under Methods).Taken together, the Ghanaian isolates were closely related; collectively, they formed a distinct phylogenetic group among genotype 2 HCV subtypes; and none of the Ghanaian HCV core sequences clustered with the reference subtypes 2a, 2b or 2c.

**Fig. 3:**
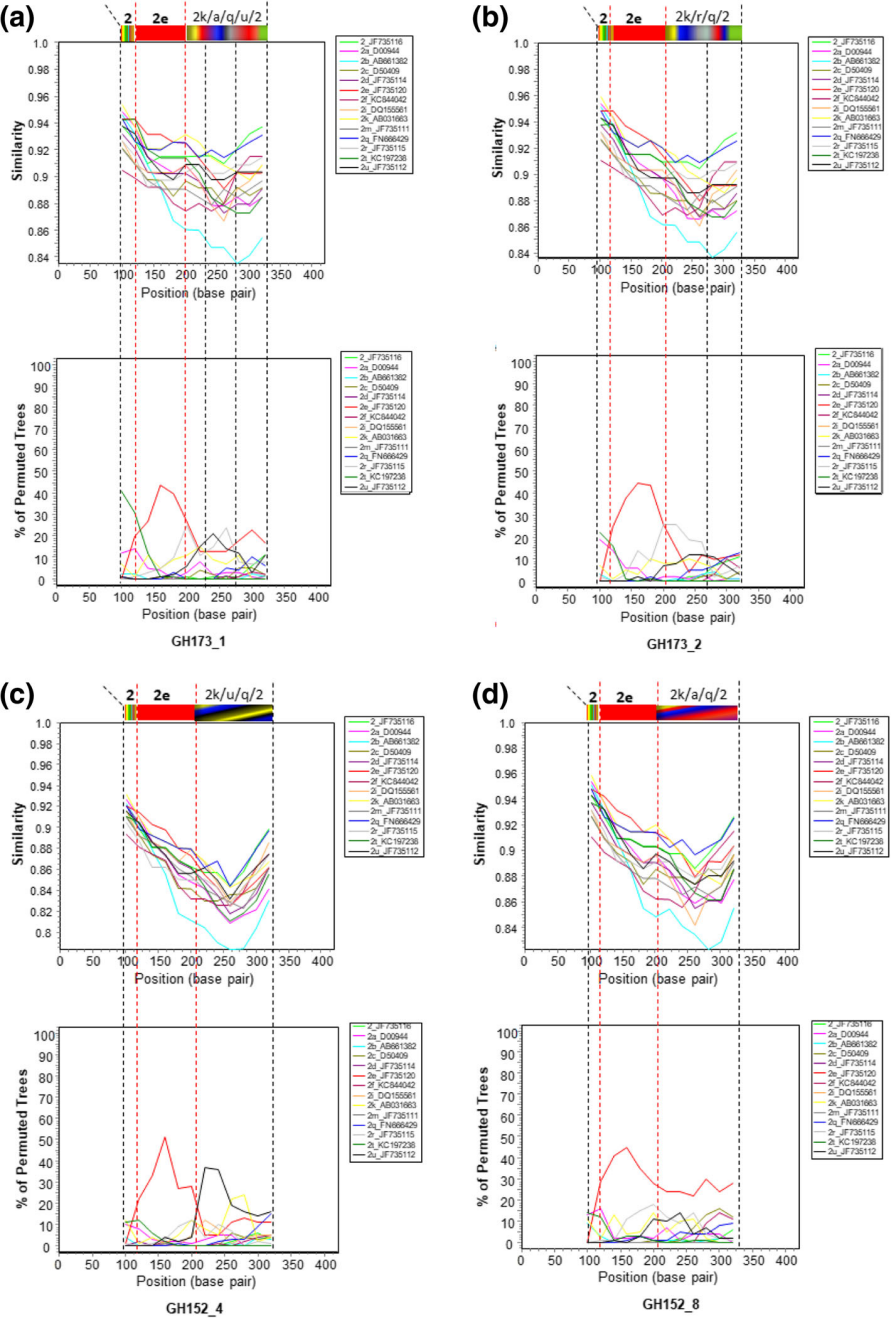
SimPlot analyses of *core* gene sequences of four Ghanaian HCV isolates. In each of the figure units (**a**), (**b**), (**c**) and (**d**), upper panel shows sequence similarity plots (SimPlot analysis) and lower panel displays bootscan analyses results. Each analysis was performed by comparing sequence relationships of test viral sequences to representatives of reference genotype 2 and its subtypes 2a, 2b, 2c, 2d, 2e, 2f, 2i, 2 k, 2 m, 2q, 2r, 2 t and 2u retrieved from the International Committee on Taxonomy of Viruses (ICTV) website (https://hcv.lanl.gov/content/sequence/NEWALIGN/align.html/). Similarity plotting and bootscan analyses were performed in SimPlot version 3.5.1 with parameters set at simple consensus sequences; and with window and step sizes of 300 and 20 nucleotides respectively. The Y-axis represents percentage of sequence similarity to the corresponding subtype in the SimPlot analysis. The bootscan panel displays plots of bootstrap values (percentage permuted trees) calculated from multiple genome alignment of test viral sequences with reference subtype sequences. Bootscanning was performed using neighbour-joining method with Kimura-2 parameter modelling for 100 replicates. For all panels the *x* axis indicates nucleotide positions along the alignment (with gaps removed from the alignment). Reference sequences used are colour-coded and listed on the right of each plot. The analyses show the presence of a unique and complex HCV genotype 2 sequence profile of the Ghanaian isolates (core gene) comprising a 2e fragment (red colour-coded) of about 80 nucleotides from position 455 to 534 of the H77 reference sequence (GenBank accession number AF009606) and other variable fragments belonging to subtypes 2a, 2 k, 2q, 2r, 2u and/or 2

To further elucidate the molecular characteristics of the HCV isolates, genomic composition of the core gene was explored in detail by similarity plotting and boot-scanning (Fig. 3). The analyses showed that, generally, the Ghanaian HCV core sequences were made up of a complex mosaic of subtype 2e fragment and other variable subtype fragments belonging to subtypes 2a, 2 k, 2q, 2r, 2u and/or 2. The approximate length of the 2e fragment was 80 nucleotides spanning positions 455 to 534 of the H77 reference sequence. Interestingly, subtype 2b appeared most distant in genetic relationship with the Ghanaian sequences. All together, these findings highlight the genetic complexity of the Ghanaian HCV sequences as far as the core gene is concerned.

## Discussion

This study investigated HCV infections and characterized viral isolates in healthy Ghanaians in Accra, the capital of Ghana. Serological screening using two standard conventional assays showed high discrepancy. Confirmation by PCR revealed low level HCV viraemia in the studied population. A previous study reported frequent recovery and low viral load in Ghanaian HCV infections [22]. Other reports also show that HCV infection in West and Central Africans rarely progresses to chronic liver disease as compared to what occurs in America, Europe and China [37–39]. Also, antibody responses to HCV are usually directed against the viral envelope proteins - most likely those derived from the hypervariable region 1 of the E2 region [40]. Therefore, extensive sequence variation of the infecting HCV strain could potentially have impact on serodiagnosis when conventional screening assays are used. As such, suitability of the assays deserves regular evaluation. Also, low viral load may explain low level viraemia detected in this study. However, the extensive polymorphism observed in HCV genome, even in conserved regions including the core, as seen in the Ghanaian samples, may give false negative results due to non-complementarity with the primer sequence used; or even a non-specific annealing of primers, which could lead to production of more than one band that may be interpreted as mixed infections. These underscore the importance of molecular characterization of HCV in different geographical areas to inform a rational design and interpretation of HCV diagnostics.

To distinguish HCV subtypes, primer-specific PCR procedure was initially applied and that classified HCV strains into subtypes under genotype 2. Sequence-based characterization by phylogenetic analysis, however, showed an identical clustering of the Ghanaian isolates under genotype 2, but the isolates exhibited high genetic variability and appeared distinct from previously described subtypes of HCV 2. Similar unique clustering pattern was observed with some previously described HCV core sequences from Ghana. Further detailed exploration of sequence patterns of the isolates by similarity plotting and bootscanning showed that the sequences were complex, having mosaic patterns of various subtypes in diverse forms. This complexity might explain their unique phylogenetic clustering and inability to assign subtypes. Thus, based on the findings of this study, the core gene sequences are not suitable for assigning subtypes of Ghanaian HCV. This adds to previous attempts at subtyping that proved unsuccessful [22, 28]. These collectively highlight the dominance of genotype 2, identical sequence clustering, extensive genetic diversity along the entire HCV genome that gives rise to complex sequence patterns and hence difficulty to distinguish subtypes of Ghanaian HCV sequences.

In a broader context, similar genetic variability and unique clustering pattern of type 2 HCV strains have been observed in neighbouring West African countries including Benin, Burkina Faso, and Cote D’Ivoire [25, 41]. Reports have also indicated that West African HCV sequences, irrespective of their geographical origin, rarely cluster with subtypes 2a to c [26, 42]. This may reflect an ongoing high rate of sequence variation in the sub-region. This may shed light on the complex nature of the Ghanaian HCV sequences described in this study. There is therefore the need for a primer update of the 5’UTR region to improve performance of the RT-PCR assay. Furthermore, primer-specific PCR-based methods that can be useful for subtyping Ghanaian HCV need to be designed and validated using information derived from Ghanaian HCV sequence profiles.

In general, high genetic variation, effective viral clearance and/or slow progression to chronicity, as commonly reported in West African HCV infections, may implicate strong immune pressure and/or effective response to HCV infection. Cellular immune responses appear to play a role in protecting against HCV infection; and possible targets for HCV-specific cytotoxic T lymphocytes (CTL) recognition epitopes have been identified within the conserved core and the highly variable E2 regions [43]. It is known that host immunogenetic factor, such as the human leukocyte antigen (HLA) system, which presents viral peptides for CTL-mediated immune response, play an important role in influencing pathogen diversity [44–47]. In fact, some HLAs have been described that associate with either clearance or persistence of HCV infection [48]. About a decade ago Chuang et al. [49] studied Ghanaian blood donors to investigate the role of cellular immune responses and host genetics in the high rate of recovery from HCV infection in West Africa. The study by Chuang and colleagues hypothesized that the dominance of genotype 2 HCV strains and an efficient contribution of HLA-B*57 may constitute important explanatory factor. It could therefore be that complexity in viral sequences as observed in this study might result from viral adaptation driven by strong HLA-mediated immune pressure on HCV. Thus, HCV heterogeneity may serve as a means of escaping CTL-mediated immunity [43, 50]. This remains to be completely defined.

Of note, HLA association with outcome of HCV infection appears to be population-specific [49, 51–53]; and immune response also appears to be HLA type-specific [54, 55], but data on these are scarce. Therefore, the seeming dynamic nature of HCV molecular evolution in Ghana, and perhaps the West African sub-region, makes it necessary to comprehensively explore the nature of immune response and the role of host genetic factors in viral diversity as well as outcome of HCV infection.

The study had some limitations. First, the number of isolates obtained for the study was low. Besides, the samples were collected only from Accra, the capital. Few samples analysed - not including samples from other regions of Ghana; and unavailability of appreciable number of HCV core sequences from neighbouring West African countries for phylogenetic comparison are some of the limitations of this study. Thus, the findings of this study may not suggest that other Ghanaian HCV-2 isolates have the same complex sequence profile. Second, specificity of the primers used for HCV detection might not be high enough. Even though published primers that had been optimized and proven to be highly specific were chosen for the test, over time, several HCV variants have evolved that might render the primers nonspecific. This could be a cause for false negative results, hence the need for primer update, especially, of the 5’UTR region to increase performance of the RT-PCR assay. Third, sequence-based HCV genotype data were obtained through direct nucleotide sequencing, which might not be sensitive enough for detecting minority HCV variants that could be obscured by the wild-type strains. Future studies should aim at initially screening a much larger number of samples, and possibly from different regions of Ghana, in order to obtain a more significant number of positive cases that can describe the genetic diversity of prevailing HCV genotypes in Ghana. Also, the use of new technology such as ultra-deep sequencing, which has higher capacity than direct sequencing in detecting both minority pathogen populations and presence of dual or multiple infections, would be very useful in analysing HCV especially in a region where the virus appears to be endemic and several subtypes may be in circulation. Nevertheless, considering the in-depth analysis conducted, and the fact that the findings of this study are largely in agreement with, and sheds light on, those reported from previous studies in other populations of Ghana and West Africa at large, it does not seem the above-stated limitations could significantly impact the results from which the study conclusions were drawn.

## Conclusions

Molecular diagnosis and characterization of HCV is very essential for the clinical management and public health control of HCV infection. This is because antigenic differences between genotypes have implications for optimal design of HCV serological assays and for development of HCV vaccines [56, 57]. As such, HCV genotype information from various geographical regions is necessary. This study found HCV seroprevalence of 1% among healthy Ghanaians in Accra; and HCV genotype 2 was the prevalent HCV type, based on analysis of the core gene. From the findings, HCV molecular epidemiology and genotype profile in Ghana appears complex. Until now HCV subtyping of Ghanaian isolates has remained a challenge; and the complexity of the core gene may not make it suitable for subtyping Ghanaian HCV strains. Further study is warranted to elucidate the HCV subtype profile and to define reliable means of subtyping Ghanaian HCV strains. An in-depth analysis of nucleotide sequences of at least two coding regions, or preferably the entire HCV genome, may yield better results. Overall, the mosaic pattern of the sequences observed in this study depict extremely high genetic diversity of the predominant genotype. This may shed some light on earlier findings that suggest that HCV genotype 2 is indigenous to Ghana [28]. The clinical implications of the high diversity of genotype 2 HCV variants in Ghana deserve further investigation.

## Data Availability

The datasets generated, used and /or analysed in this study are accessible from public database (DDBJ) and/or from NIN and AKN on reasonable request.

## Abbreviations

5’UTR 5′: Untranslated region (Five prime untranslated region)
CTL: Cytotoxic T lymphocytes
DEPC: Diethyl pyrocarbonate
DNA: Deoxyribonucleic acid
dNTP: Deoxyribonucleotide triphosphate
E1/E2: Envelope gene 1 or gene 2
EDTA: Ethylenediaminetetraacetic acid
ELISA: Enzyme-linked immunosorbent assay
HCV: Hepatitis C virus
HLA: Human leukocyte antigen
MEGA: Molecular Evolutionary Genetics Analysis
M-MuLV: Moloney murine leukemia virus
mRNA: messenger RNA
NS5B: Non-structural 5B gene
PA: Particle agglutination
RNA: ribonucleic acid
RT-PCR: Reverse transcription polymerase chain reaction
TAE: Tris-acetic ethylenediaminetetraacetic acid
WHO: World Health Organization
NMIMR: Noguchi Memorial Institute for Medical Research
JICA: Japan International Cooperation Agency
ICTV: International Committee on Taxonomy of Viruses
DDBJ: DNA Data Bank of Japan
